# Review of the Potential Role of Ascorbate in the Prevention and Treatment of Gynecological Cancers

**DOI:** 10.3390/antiox13050617

**Published:** 2024-05-19

**Authors:** Xiaochang Shen, Jiandong Wang, Boer Deng, Ziyi Zhao, Shuning Chen, Weimin Kong, Chunxiao Zhou, Victoria Bae-Jump

**Affiliations:** 1 Department of Gynecologic Oncology, Beijing Obstetrics and Gynecology Hospital, Capital Medical University, Beijing Maternal and Child Health Care Hospital, Beijing 100006, China; xcshen96@ad.unc.edu (X.S.); wangjiandongxy@ccmu.edu.cn (J.W.); brdeng27@email.unc.edu (B.D.); zhaoziyi@email.unc.edu (Z.Z.); csn2023@email.unc.edu (S.C.); kwm1967@ccmu.edu.cn (W.K.); 2 Division of Gynecologic Oncology, University of North Carolina at Chapel Hill, Chapel Hill, NC 27599, USA; 3 Lineberger Comprehensive Cancer Center, University of North Carolina at Chapel Hill, Chapel Hill, NC 27599, USA

**Keywords:** ascorbate, gynecological cancer, prevention, treatment

## Abstract

Ascorbate (vitamin C) is an essential vitamin for the human body and participates in various physiological processes as an important coenzyme and antioxidant. Furthermore, the role of ascorbate in the prevention and treatment of cancer including gynecological cancer has gained much more interest recently. The bioavailability and certain biological functions of ascorbate are distinct in males versus females due to differences in lean body mass, sex hormones, and lifestyle factors. Despite epidemiological evidence that ascorbate-rich foods and ascorbate plasma concentrations are inversely related to cancer risk, ascorbate has not demonstrated a significant protective effect in patients with gynecological cancers. Adequate ascorbate intake may have the potential to reduce the risk of human papillomavirus (HPV) infection and high-risk HPV persistence status. High-dose ascorbate exerts antitumor activity and synergizes with chemotherapeutic agents in preclinical cancer models of gynecological cancer. In this review, we provide evidence for the biological activity of ascorbate in females and discuss the potential role of ascorbate in the prevention and treatment of ovarian, endometrial, and cervical cancers.

## 1. Introduction

Ascorbate (C_6_H_8_O_6_-), also known as vitamin C, is a water-soluble vitamin consisting of a skeleton of six-carbon lactone derived from fruits and vegetables [1]. As it cannot be synthesized within the human body, daily diet or iatrogenic routes are the only ways for people to obtain ascorbate [1,2]. The physiological function of ascorbate is primarily based on its ability to alter the redox state and the subsequent transfer of electrons, finally converting ascorbate into its oxidized form, dehydroascorbic acid (DHA), an unstable molecule with a half-life of about 6 min [1,3]. DHA is easily hydrolyzed to diketogulonate and loses its activity of ascorbate outside the cells, or it may be transported into cells and then rapidly reduced to ascorbate again in the cytoplasm, which partially helps maintain the normal concentration of plasma and cellular ascorbate [3]. Notably, the stable physiological concentrations of plasma ascorbate are essential to maintaining its physiological functions by acting as a cofactor of various oxygenases and participating in various essential physiological processes, acting as a scavenger to eliminate potentially harmful oxidizing free radicals to protect tissues and cells from oxidation, and serving as a reducing agent to increase intestinal iron absorption [1,4]. Overall, ascorbate is an essential nutrient involved in homeostasis maintenance, cell division and growth, signal transduction, immune function modulation, immune barrier establishment, and disease prevention in the body.

The history of ascorbate being used as a medicine for scurvy treatment dates back more than 300 years [5]. Following the successful isolation and synthesis of ascorbate in the early 20th century, scientists identified more physiological functions of ascorbate in the body and found even more diseases that could be cured or improved by taking ascorbate [6]. Ascorbate has been investigated as an antitumor drug in preclinical models and clinical trials since the 1970s. However, inconsistent results have led to questions about its antitumor properties in clinical trials [7,8,9]. Until the end of the 20th century and with a deepening understanding of the distribution of pharmacokinetics, ascorbate was found to inhibit cancer cell proliferation and tumor growth in almost all preclinical models without obvious side effects when the concentrations of ascorbate reached the levels of millimoles, called high-dose ascorbate [10]. The level of high-dose ascorbate can only be achieved by the parenteral route including intravenous (IV) and intraperitoneal (IP) routes in vivo due to oral ascorbate being susceptible to the intestinal environment, with multiple phase I and II clinical trials already demonstrating its safety in cancer patients [11,12]. The underlying mechanisms of high-dose ascorbate revolve around its pro-oxidant activity as opposed to antioxidant activity, thereby disrupting the redox balance in tumor cells and causing lethal effects on tumor cells [5,13]. Over the past three decades, studies on the antineoplastic effects of ascorbate as a single agent or in combination with other chemotherapeutic agents have grown substantially in various cancers, including gynecological cancers [14,15,16,17,18]. These results indicate that high-dose ascorbate exhibits potent antitumor activity, as well as synergy with chemotherapeutic drugs in various cell and animal models of cancer. 

Gynecological cancer is any type of cancer originating from the female reproductive system, including ovarian cancer; endometrial cancer; cervical cancer; vaginal cancer; vulva cancer; and other rare female genital malignancies, such as fallopian tube carcinoma. It is estimated that there will be 116,930 new cases of gynecological cancer and 33,850 fatalities resulting from the diseases in the United States in 2024, with cervical cancer, ovarian cancer, and endometrial cancer accounting for over 85% of new cases and over 90% of deaths, respectively [19]. While gynecological cancers are connected to reproduction in terms of function, they involve distinct tumor biology and exhibit a diverse array of risk factors and epidemiological characteristics [20,21,22]. The approaches to managing and preventing gynecological cancers vary subtly depending on the distinct biological properties of gynecological cancers. Current treatment strategies such as surgery, chemotherapy, and radiotherapy have effectively improved the survival of most patients with gynecological cancers, but there are still many advanced and recurrent patients who do not benefit from these treatments [20,21,22]. As a result, innovative approaches to disease prevention and treatment may help reduce the burden of gynecological cancer. This review will introduce the unique bioavailability of ascorbate in females and discuss the possibility of ascorbate as a strategy in the prevention and treatment of gynecological cancers.

## 2. Bioavailability of Ascorbate in Females

Given that human beings do not have a functional gene for L-gulono-γ-lactone oxidase, which catalyzes the terminal step of ascorbate biosynthesis, the supplementation of ascorbate required by the human body relies on exogenous sources [1]. Adequate exogenous intake, efficient absorption, metabolism, and excretion of ascorbate are vital for human beings to maintain ascorbate plasma levels. The main dietary sources of ascorbate are plant species, including black currant; kiwi; strawberry; and cruciferous vegetables, especially broccoli, kale, and peppers [23]. The absorption of ascorbate in the human body is mostly in the distal ileum and mediated by the sodium-dependent vitamin C transporter 1 (SVCT1), a sodium-dependent transporter that moves ascorbate with a Km of approximately 70 µM, which is highly responsible for the maintenance of ascorbate levels throughout the body [24]. SVCT1 is produced by the Slc23a1 gene located at chromosome locus 5q31.2–1.3 and is highly expressed in epithelial tissues, such as the small intestine, skin, kidney, liver, pancreas, lung, and reproductive organs [24,25]. The saturable and sodium-dependent characteristics of SVCT1 make the absorption of ascorbate not linear, and the peak plasma concentrations of oral ascorbate do not exceed 220 µM regardless of the dosage of ascorbate [1]. Ascorbate is not bound to any proteins in plasma after absorption, and a portion of ascorbate is transported into tissue cells via sodium-dependent vitamin C transporter 2 (SVCT2), a close analog of SVCT1 that is produced by the gene of Slc23a2 at the chromosomal locus 20p12.2–12.3 [26]. SVCT2 mediates electrochemical sodium gradient-reliant unidirectional ascorbate transport with a Km of 15 µm and is ubiquitous present in the majority of metabolically active tissues, including the brain (neurons), retina, bone, heart, lung, placenta, spleen, prostate, testis, ovary, skeletal muscle, kidney, and adrenal gland, all of which maintain higher intracellular and extracellular fluid concentrations of ascorbate than plasma and protects the tissue from oxidative stress [24,25]. The excretion of ascorbate is substantially through the kidney, and less than 1% is excreted with the feces [1]. Additionally, because of the high expression of SVCT1 in the brush border of the proximal tubule of the kidney, renal reabsorption of ascorbate is an effective means of maintaining the concentration of plasma ascorbate [24,27]. Knockout of the Slc23a1 gene markedly reduced ascorbate levels in plasma and tissues in mice and triggered a more than 18-fold increase in ascorbate excretion, resulting in up to a 70% reduction in ascorbate body stores through urine daily, suggesting that SVCT1 is a dominant transporter in maintaining the delicate balance of plasma ascorbate in the body [27]. 

**Figure 1:**
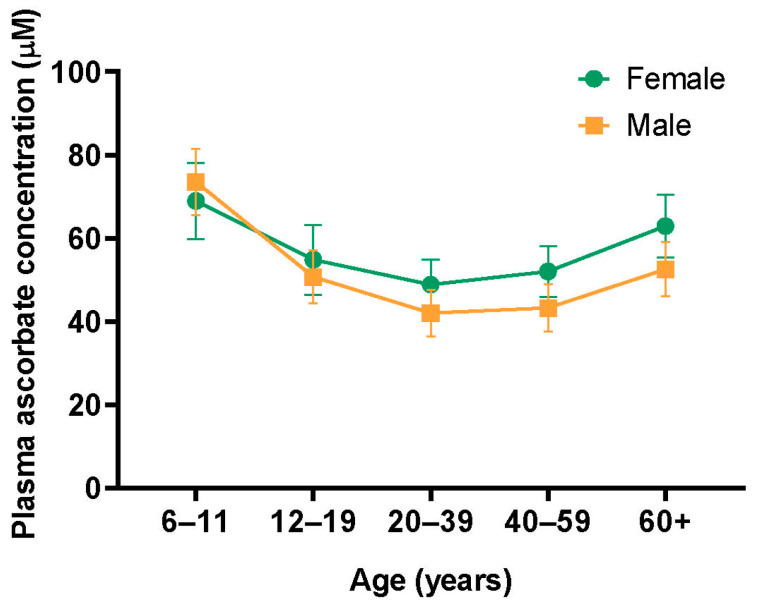
Fluctuations in plasma ascorbate concentration over the lifespans in males versus females. Females (Green) showed varying levels of plasma ascorbate concentration throughout their lifespan, reaching peak levels between the ages of 6 and 11, decreasing as they aged, and then rising slowly after reaching 40. Males (Yellow) displayed a comparable tendency to females, with lower plasma ascorbate concentrations than females at equivalent ages [29].

As a foundational nutrient for the body, the recommended diet allowance (RDA) for ascorbate is up to 90 mg/day for males and 75 mg/day for females. The RDA for ascorbate is variable in the lifetime of females, from childhood to menopause, according to the Institute of Medicine in the United States (Table 1) [28]. Females generally have a 15–30% higher concentration of plasma ascorbate than males after adolescence [29,30]. The difference in the ingestion of ascorbate between males and females cannot fully explain the differences between sexes in the concentrations of plasma ascorbate, as the concentrations of plasma ascorbate in females remain higher when consuming similar amounts of ascorbate as in males [29,31]. Current data support that the difference between the sexes is partly due to a volumetric dilution effect, as females have lower fat-free mass (FFM), causing lower distribution volumes and, therefore, higher circulating concentrations of ascorbate when compared to males [31,32]. The National Health and Nutrition Examination Survey conducted in the United States provides further support for the FFM theory through its observation of fluctuations in plasma ascorbate levels over the lifespans of males versus females (Figure 1). This study demonstrated that the highest concentrations of plasma ascorbate were found in females aged 6–11, and a gradual decline in levels was observed with increasing age, with a slow increase after the age of 40, which also occurred in males [29]. The fluctuation is contrary to changes in FFM in females over their lifespan, as FFM between 8 and 18 years of age gradually increases, stabilizes at the ages of 18–39, and decreases after the age of 40 [29,33,34]. However, the differences in FFM only accounted for approximately 30% of the variation in plasma ascorbate parameters [35]. Numerous animal and clinical studies have confirmed that alternations in estrogen and progesterone in females also contribute to the difference. Administration of estrogen or progesterone alone for over two weeks significantly reduced plasma ascorbate concentrations in rats and guinea pigs [36,37]. The long-term use of oral contraceptives (OC) can significantly decrease circulating ascorbate concentrations in females by up to 40% [38,39]. After oral supplementation with 300 mg ascorbate, OC users had a lower increase in plasma ascorbate levels than non-OC users, further confirming the role of exogenous estrogen and progesterone in modulating plasma ascorbate levels [39]. 

There are currently no specific mechanisms elucidating how estrogen and progesterone modulate the concentrations of plasma ascorbate in females. Possible explanations include the oxidative effects of estrogen and progesterone, leading to increased degradation of ascorbate, the restoration of fluid balance, and changes in tissue uptake patterns [38,39,40,41]. Additionally, circulating ascorbate concentrations were found to vary regularly throughout the menstrual cycles, suggesting that endogenous female hormones are also involved in the regulation of plasma ascorbate levels, although there are conflicting results due to factors such as the investigated population, detection timing, ascorbate intake pattern, and analytical procedures [38,40,42]. Lower ascorbate excretion in female mice may contribute to higher plasma concentrations of ascorbate, but no sex-related differences were found in ascorbate renal excretion and maximum tubular reabsorption in a similar human study [43,44]. The disparities in lifestyle between males and females, including higher levels of smoking and alcohol consumption amongst males, may also contribute to the difference in plasma ascorbate [29,45]. Smoking, alcohol consumption, and exogenous stressors have the capacity to induce an increase in free radicals and trigger a higher ascorbate metabolic turnover, resulting in the potential to biochemically reduce the concentration of plasma ascorbate in males [29].

Pregnancy and breastfeeding stages also change the RDA, increasing by 10 mg/day and 45–50 mg/day, respectively, on the basis of the ascorbate requirement for non-pregnancy, to meet the needs of fetuses and newborns [28] (Table 1). Ascorbate is essential for normal fetal growth and development as the fetus relies completely on maternal supplements through SVCT2 transportation in the placenta [27,46]. A two-thirds drop in plasma ascorbate levels in female mice resulted in a substantial rise in perinatal mortality in pups, while oral ascorbate supplementation in female mice effectively reduced pup mortality [27]. Moreover, retrospective studies demonstrated that the deficiency of ascorbate in late pregnancy led to a higher risk of preterm premature rupture of membranes and placental abruption [47,48]. A recent randomized controlled clinical trial proved the efficiency and safety of ascorbate supplementation in females with a history of premature rupture of membranes [49]. Overall, as an essential vitamin, the requirements of ascorbate and the fluctuations in plasma level vary during the female life cycle. FFM changes, sex hormones, and varying lifestyle choices may be factors that contribute to the difference in ascorbate concentrations between males and females, and the prevention of ascorbate deficiency is critical to the health of females during their lifetime, especially during pregnancy.

## 3. Physiological Functions of Ascorbate

**Figure 2:**
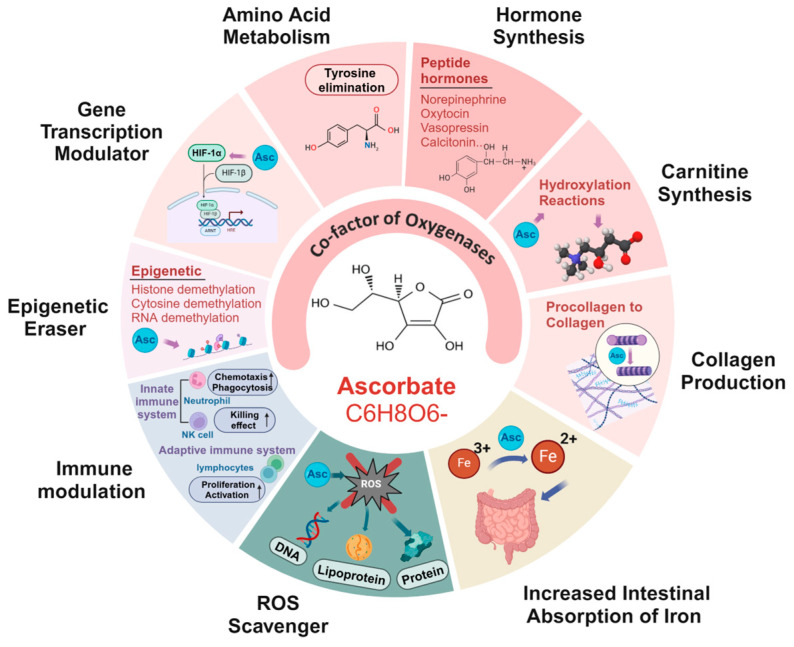
Physiological functions of ascorbate (Asc). Ascorbate plays distinct physiological functions in the body. Its primary function is to serve as a cofactor for over 20 types of oxygenase and to participate in various fundamental physiological processes, including collagen synthesis, carnitine production, hormone synthesis, amino acid metabolism, gene regulation, and epigenetic modification. Ascorbate acts as a scavenger to neutralize various harmful reactive oxygen species, thereby protecting nucleic acids, proteins, and lipids from oxidative damage. Furthermore, ascorbate increases the absorption of iron in the intestines by reducing ferric iron (Fe^3+^) to ferrous iron (Fe^2+^), marking it an effective iron absorption enhancer. Ascorbate also acts as an antioxidant and cofactor to protect immune cells and enhance innate and adaptive immune functions.

The prominent biological role of ascorbate is as an enzyme cofactor for more than 20 oxygenases by virtue of its electron-donating properties that participate in various physiological processes (Table 2) [1]. Apart from being a cofactor, ascorbate is also recognized for its ability to effectively scavenge various reactive oxygen species, such as copper ions and iron, and to protect important biomolecules, including nucleic acids, proteins, and lipids, from oxidant damage resulting from endogenous metabolism and exogenous toxins in the human body [1,50]. By reducing ferric iron (Fe^3+^) to ferrous iron (Fe^2+^), ascorbate can enhance intestinal iron absorption and increase the bioavailability of iron [1,4]. This makes ascorbate the most efficient enhancer of iron absorption, especially important for preventing iron deficiency and iron deficiency anemia in females of reproductive age [4,51,52]. In addition, ascorbate serves as a potent antioxidant and cofactor involved in protecting lymphocytes from oxidative damage and modulating the activity of biosynthetic and gene-regulating enzymes in the innate and adaptive immune systems, thereby reducing susceptibility to infections [53,54] (Figure 2).

## 4. Ascorbate in Gynecological Cancers

**Figure 3:**
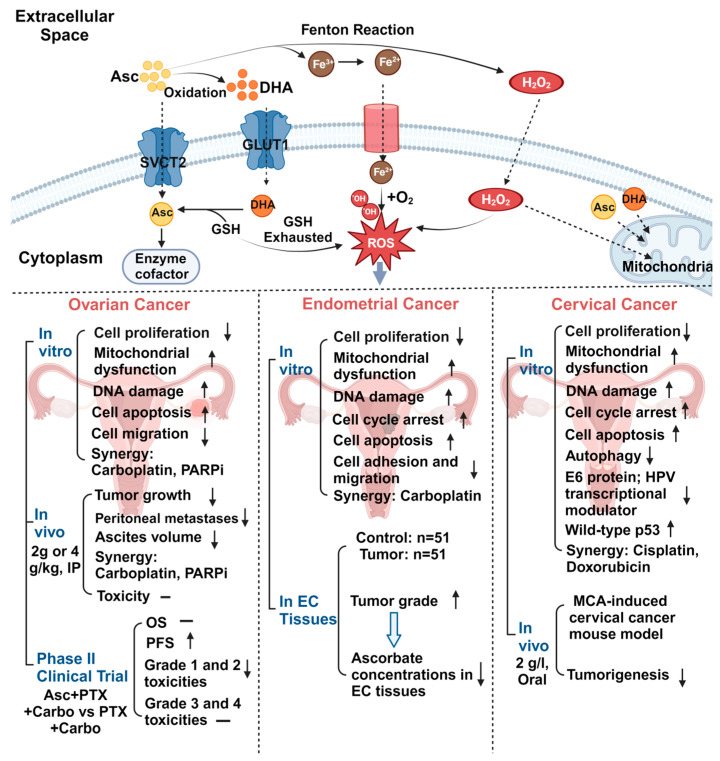
The antitumor activity of high-dose ascorbate (Asc) in gynecological cancers. With the catalysis of Fe^3+^, high-dose extracellular ascorbate generates substantial H_2_O_2_ and enhances Fe^2+^ uptake, initiating the Fenton reaction and continuously generating ROS, which eventually leads to cell death. Extracellular Asc can be transported into the cell through SVCT2 or converted to DHA and enter cells via GLUT1. Within cells, DHA can rapidly be reduced back to Asc by reduced glutathione (GSH), depleting GSH, and increasing ROS. In the cytoplasm, high-level Asc serves as a cofactor of enzymes to affect key molecular regulators such as HIF-1α, TET, and JHDM. Hence, high-dose ascorbate demonstrated potent antitumor activities in ovarian cancer, endometrial cancer, and cervical cancer. (↑: increase, ↓: decrease, -: no significant change, EC: endometrial cancer).

Gynecological cancers pose a threat to females’ health and longevity, accounting for 12.1% of estimated new cancer cases and 11.7% of cancer-related deaths in the United States in 2024, preceded only by breast cancer, digestive system cancers, and respiratory system cancers [19]. The treatment of these malignant cases varies according to the source and type of tumor and primarily includes surgery, chemotherapy, radiotherapy, immunotherapy, and targeted therapy [20,21,22]. Regardless of substantial advances in the treatment of early-stage gynecological cancers over the past few decades, the prognosis of patients with advanced, recurrent, and metastatic disease remains dismal [69]. Exploring new therapy strategies is one of the most reliable ways to improve the therapy outcomes and survival of these patients. Since 1969, studies have consistently shown that high-dose ascorbate in a range of 0.5–20 mM has antiproliferative and antimetastatic activities in various types of cancer cells in a dose-dependent manner without influencing the viability of normal cells or even showing protective effects [1,12,70]. High-dose ascorbate (1–4 g/kg, IP) administration once or twice a day for 21–45 days demonstrated potent growth inhibition and a reduction in metastatic foci in multiple xenograft models, PDX models, and transgenic models of cancer, with a favorable safety profile [11,15,16,71,72,73]. The combination of high-dose ascorbate with various chemotherapeutic drugs has been found to have synergistic effects on cell cycle dysregulation, the activation of both extrinsic and intrinsic apoptotic pathways, and the induction of DNA damage levels compared with single drug treatment [12,16,74,75]. Recent clinical trials found that high-dose IV infusions of ascorbate (1.5–2.2 g/kg, 3× weekly, over 6 weeks) combined with chemotherapeutic agents improved progression-free survival (PFS) or overall survival (OS) in some types of cancer without serious side effects [16,72,76,77]. Under the catalysis of endogenous or exogenous metals such as Fe^3+^, high-dose ascorbate produces large amounts of H_2_O_2_ and increases the cellular stress levels, resulting in DNA damage, cell cycle arrest, apoptosis, and autophagy while inhibiting epithelial–mesenchymal transition (EMT) and angiogenesis [5,12,18,71,78]. Therefore, it is currently believed that the antitumor mechanism of ascorbate mainly utilizes its pro-oxidative activity rather than antioxidative activity in most cancer cells [70]. The characteristic of ascorbate as a cofactor for oxygenases also makes ascorbate take part in genetic and epigenetic regulation to affect cell survival [79,80]. The protective effect of ascorbate in normal cells is likely due to the low levels of redox-active labile metals and high levels of antioxidant enzymes including catalase and glutathione peroxidase to eliminate the effect of H_2_O_2_ [13,81]. Given the potent antitumor activity shown by ascorbate in various preclinical studies and its unique association with females’ health, the role of ascorbate in gynecological cancers, including in pathogenesis, treatment, and prevention, is gradually gaining importance. Recent results support that ascorbate exhibits antiproliferative and anti-invasive activities in gynecological cancers through its pro-oxidative activity (Figure 3).

### 4.1. Ovarian Cancer

Ovarian cancer is the most lethal gynecological cancer, with a five-year relative survival below 45% [20,82]. Initial therapy for ovarian cancer includes debulking surgery and platinum-based chemotherapy in combination with paclitaxel. Recently, antiangiogenic agents and poly(ADP ribose) polymerase (PARP) inhibitors have been incorporated into the ovarian cancer treatment paradigm [82,83]. However, chemoresistance is a major obstacle affecting the efficacy of treatment for ovarian cancer; thus, there is an urgent need to develop new therapeutic management strategies for these patients [20,82].

#### 4.1.1. The Effect of Ascorbate in the Prevention of Ovarian Cancer

Since oxidative stress is involved in the carcinogenesis of ovarian cancer through genetic alternations, the modulation of signaling pathways, the modification of transcription factors, and changes in the tumor microenvironment, multiple epidemiological studies have investigated the role of ascorbate in the prevention of ovarian cancer [84,85]. A case–control study including 419 cases using a food frequency questionnaire (FFQ) investigated ascorbate intake in the year before the date of diagnosis for cancer cases and in the year before the date of the interview for control cases [86]. The results showed that a higher intake of ascorbate (>363 mg/day) was associated with a 45% lower risk of ovarian cancer (odds ratio (OR) = 0.45) [86]. Another case–control study using an FFQ in 254 patients and 652 controls to measure ascorbate consumption in the five years before the diagnosis of ovarian cancer also revealed that ascorbate reduced the risk of ovarian cancer in the group with the highest ascorbate consumption (>140.25 mg/day) compared with the group having the lowest consumption (<66.50 mg/day) (OR = 0.31) [87]. Furthermore, a hospital-based prospective cohort study of ovarian cancer patients aged 18 to 79 between 2015 and 2020 found that higher ascorbate dietary intake (one year before diagnosis) based on a 111-item self-administered FFQ was associated with improved ovarian cancer survival after a median of 37.19 months of follow-up (hazard ratio (HR) = 0.43) [88]. However, another FFQ-based prospective cohort study followed 97,275 females in the California Teachers Study cohort for eight years and found higher dietary ascorbate intake (>665 mg/day) could significantly increase the risk of ovarian cancer by 150% compared to the group with the lowest ascorbate intake (≤75 mg/day) [89]. Several case–control and prospective studies have found no correlation between ascorbate consumption and the risk of ovarian cancer [90,91,92]. A pooled analysis of 10 cohort studies including 501,857 females with a 7–16-year follow-up found no relationship between ascorbate intake and the risk of ovarian cancer both in dietary and supplement sources [93]. Since most of these studies are case–control studies, the results are likely to be affected by recall bias and selection bias [89,92]. Additionally, a variety of confounding variables in these studies, including population makeup, geographic locations, dose and source of ascorbate, and the complex biological interactions between multiple nutrients in the daily diet, may have confounded these results [92].

#### 4.1.2. The Antitumor Role of Ascorbate in Ovarian Cancer

The inhibition of cell proliferation by high-dose ascorbate has been reported in seven human ovarian cancer cell lines (A2780, OVCAR3, OVCAR5, OVCAR8, OVCAR10, SKOV3, and SHIN3) and one mouse ovarian epithelial papillary serous adenocarcinoma cell line (ID8) with IC50s of 0.3 to 3 mM, whereas ascorbate treatment failed to significantly inhibit the proliferation of immortalized nontumorigenic ovarian epithelial cells [16,94,95]. The cytotoxicity induced by high-dose ascorbate in ovarian cancer cells can be effectively reversed by catalase, a specific H_2_O_2_ scavenger, suggesting the crucial role of oxidative stress in ascorbate-induced cell death in ovarian cancer cells [16,94,96]. Treatment of ovarian cancer cell line SHIN3 with ascorbate activated the protein ataxia–telangiectasia mutated (ATM) kinase, a key mediator of cellular response to DNA damage, through the activation of AMP-activated protein kinase (AMPK) and the inhibition of the mechanistic target of rapamycin (mTOR) pathways [16,94]. In ID8 mouse ovarian cancer cells, treatment with 1.5 mM ascorbate for 24 h caused the loss of mitochondrial membrane potential, increased calcium overload, and activated cleaved caspase-3 [95]. Parenteral administration of ascorbate (4 g/kg, twice a day) also significantly reduced tumor weight by 50% in the OVCAR5 subcutaneous xenograft mouse model [96].

Different from hematogenous metastasizing tumors, ovarian cancer cells primarily disseminate within the peritoneal cavity and omentum via peritoneal fluid [97]. Ascorbate at a dose of 2 mM for 24 h significantly inhibited the spheroid formation and wound healing ability of ID8 mouse ovarian cancer cells [95]. Treatment of ascorbate (2 g/kg or 4 g/kg, IP, twice a day, 6 weeks) in an ID8 intraperitoneal xenograft mouse model dose-dependently reduced the number of tumor-associated macrophages, decreased the number of spherocytes, and induced the disruption of ID8 spheroid structures [95]. Furthermore, ascorbate (4 g/kg, IP, twice a day) treatment for 25 days significantly inhibited intraperitoneally implanted tumor burden and ascites formation without affecting body weight and causing pathological changes in other organs such as liver, kidney, and spleen, as detected by H&E staining in the intraperitoneal xenograft mouse models of ovarian cancer [16,94]. Overall, these results indicate that ascorbate can inhibit peritoneal metastases in mouse models of ovarian cancer, although the mechanism needs to be further explored.

PARP inhibitors, as a novel class of antitumor agents that disrupt single-strand DNA breaks, are already approved for the frontline maintenance therapy of recurrent ovarian cancer [98]. Unfortunately, some breast cancer gene (BRCA)1/2 wild-type ovarian cancer patients still show low responsivity to PARP inhibitors, limiting their clinical efficacy [98]. Combinations of 2.5 mM ascorbate with either olaparib (20 µM) or veliparib (20 µM) significantly decreased cell viability and the expression of BRCA1 and BRCA2 compared to single-agent treatment in the BRCA1/2 wild-type OVCAR5 and SHIN3 cell lines [94]. Furthermore, the combination treatment of mice with ascorbate (4 g/kg, IP) and olaparib for 25 days showed greater inhibition of tumor weight and ascites volume compared with single-drug treatment in intraperitoneal xenograft mouse models of ovarian cancer [94]. These results suggest that ascorbate can overcome the resistance of BRCA1/2 wild-type ovarian cancer cells to olaparib. Primary and acquired platinum resistance remains a major clinical challenge in advanced or recurrent ovarian cancer [99]. The combination of ascorbate and carboplatin exhibited greater inhibition of cell proliferation in the OVCAR5, OVCAR8, and SHIN3 cell lines and tumor growth inhibition in an intraperitoneally implanted SHIN3 cell xenograft model compared with a single agent alone, which was accompanied by a significantly enhanced DNA damage levels in the combination group [16]. In a phase I/IIa clinical trial, 25 patients with newly diagnosed stage III or IV ovarian cancer were treated with ascorbate (IV, 75 or 100 g per infusion, 2× weekly, 12 months) plus carboplatin/paclitaxel [16]. The doses of ascorbate were previously proven to maintain plasma ascorbate levels above 10 mM for several hours in other clinical trials [72,100]. The results showed a prolonged PFS of 8.75 months and a trend toward OS compared with the chemotherapy-only group, without increasing the rate of grade 3 or 4 toxicity and importantly decreasing grade 1 and 2 toxicity according to the NCI Common Terminology Criteria for Adverse Events, Version 3.0 (CTCAE v3) [16].

### 4.2. Endometrial Cancer

Endometrial cancer is the most common gynecological cancer, with 67,880 estimated new cases and 13,250 estimated cancer-related deaths in the United States in 2024 [19]. Obesity is one of the most recognized risk factors for the carcinogenesis and development of endometrial cancer, and the increasing prevalence of obesity has led to a progressive increase in the incidence of endometrial cancer [21]. The obesity-associated chronic inflammatory state and altered hormonal balance contribute to the development and progression of endometrial cancer [101]. Thus, it is of interest whether the anti-inflammatory effects of ascorbate may be of benefit in the prevention of endometrial cancer.

#### 4.2.1. Ascorbate in the Prevention of Endometrial Cancer

Insufficient ascorbate levels in the body caused by obesity and obesity-induced chronic inflammation may impair antioxidant defenses and increase the risk of oxidative stress-related complications, such as cardiovascular diseases and cancers [102,103]. A systematic literature review and meta-analysis published in 2008, including 1 cohort study and 10 case–control studies involving 5004 endometrial cancer cases and 12,746 healthy controls, was the first to show that higher intakes of ascorbate (50 mg/1000 kcal) modestly reduced the risk of endometrial cancer by 15% [104]. However, a recent Mendelian randomization study analyzed the genetic data from 12,906 endometrial cancer cases and 108,979 controls for 11 single-nucleotide polymorphisms (SNPs) robustly associated with plasma circulating ascorbate and genetically predicted that plasma ascorbate concentration increased by 20 μM was causally associated with a 37% higher endometrial cancer risk in the European descent population (OR = 1.374) [105]. This same effect was observed when dividing these cases into endometrioid (OR = 1.324, *p* = 0.0881) and non-endometrioid (OR = 1.392, *p* = 0.1647) subgroups [105]. In contrast, a cross-sectional study using two consecutive 24 h dietary recall-based FFQs in 12,437 females found no relationship between ascorbate intake and the risk of endometrial cancer [106]. Similar results have also been shown in other case–control studies [106,107,108]. The reason for such contradictory results is that, in addition to the common confounding variables such as population makeup and geographic location and the different types of data, most studies did not include body mass index, an independent risk factor of endometrial cancer and a negative regulator of plasma ascorbate concentrations, in the analysis.

#### 4.2.2. The Antitumor Role of Ascorbate in Endometrial Cancer

Few studies have addressed the antitumor activities of ascorbate in endometrial cancer. In 1989, Noto et al. first discovered that ascorbate in the range of 0.5–10 mM produced a significant growth inhibitory effect on the endometrial cancer cell line AN3CA in a dose-dependent manner [17]. The simultaneous incubation of catalase with ascorbate for one hour completely reversed the effect of ascorbate on growth inhibition in AN3CA cells [17]. In 2010, a study found that ascorbate concentrations gradually decreased with increasing tumor grade, with grade 3 tumors containing approximately 40% less ascorbate than matched adjacent normal tissues [109]. Poor vascularization and low oxygen levels in high-grade tumors and reduced sodium-dependent vitamin C transporter (SVCT) activity in acidic environments may be responsible for the low level of ascorbate in tumor tissues [109]. Our recent study in uterine serous carcinoma, a subtype of endometrial cancer, found that the millimole level of ascorbate significantly inhibited cell proliferation, induced DNA damage and apoptosis, and decreased cell adhesive and migratory ability through the cell stress pathway. The combination of ascorbate with carboplatin demonstrated synergistic effects via the induction of cellular stress, DNA damage, and apoptosis in uterine serous carcinoma cells [110]. However, whether ascorbate treatment in endometrial cancer can inhibit tumor growth has not yet been tested in preclinical animal models.

### 4.3. Cervical Cancer

Cervical cancer is the fourth most common cancer in women worldwide and is a major global health challenge, especially in low- and middle-income countries where the mortality is 18 times higher than in developed countries [22]. High-risk subtypes of HPV are associated with virtually all cases of cervical cancer [22,111]. Since ascorbate has been shown to be a potential drug for preventing and alleviating infections caused by bacteria and viruses, this has raised more interest in the antivirus role of ascorbate in the carcinogenesis and development of cervical cancer [112,113].

#### 4.3.1. Ascorbate in the Prevention of Cervical Cancer

Increasing evidence has clearly confirmed that HPV infection leads to the development of cervical intraepithelial neoplasia (CIN), and high-risk HPV infection is the main risk factor for the subsequent evolution of cervical squamous cell carcinoma and adenocarcinoma [22,111]. Although HPV infection is transient in most females, it is not cleared in 10–20% of infected females, resulting in persistent infection and eventually leading to the development of cervical cancer [114]. Given that high plasma antioxidant concentrations appear to be protective against HPV infection in females, several studies have investigated the role of plasma ascorbate concentrations or dietary intake of ascorbate in females with HPV infection [115]. A recent cross-sectional study of 2174 females aged 18–59 years in the United States found that higher serum ascorbate levels were associated with lower rates of HPV infection in females aged 25–59 years, but no association was found in those under 25 years of age, possibly due to the high prevalence of HPV infection in younger female and the high rate of auto-clearance after HPV infection [116]. Another study involving 433 females from low-income families in Brazil demonstrated that those females with higher ascorbate intake had a lower risk of persistent HPV infection over a 12-month period of follow-up after adjustment for other non-nutrient factors [117]. A similar study found that high-risk HPV-positive females had a lower ascorbate intake than uninfected females [118]. These results indicate that dietary intake of high concentrations of ascorbate may reduce the risk of HPV and alter the status of persistent HPV infection.

Ascorbate has been found to enhance the mucosal immune response to HPV infection, serve as an effective scavenger of free radicals and oxidants, inhibit DNA adduct formation, and decrease the frequency of genomic translocations [119]. Thus, it is biologically plausible that ascorbate may protect against cervical carcinogenesis [1,53,115,119]. Patients with CIN demonstrate decreased antioxidants in their plasma and increased oxidative stress in their erythrocytes, suggesting that ascorbate may have potential protective beneficial effects through its antioxidant effects [120,121]. A case–control meta-analysis comprising publications between 1986 and 2008 found that ascorbate intake or other antioxidants, including vitamin E, vitamin B12, and beta-carotene, had a significant protective effect against CIN and invasive cervical cancer [122]. Similar results were found in another meta-analysis published in 2016, which showed that ascorbate intake was significantly associated with the reduced risk of cervical neoplasia (CN), and increased ascorbate intake of 50 mg/day reduced the risk of CN by 15% [123]. Interestingly, geographic region appears to be one of the major factors influencing the effects of ascorbate and the risk of CN [123]. Given the potential bias of case–control studies, prospective cohort studies and randomized clinical trials (RCTs) may provide more clarity on the effects of ascorbate in the carcinogenesis and progression of cervical cancer. However, two recent cohort studies have yielded controversial results. A newly published study of 2304 Chinese females found that lower ascorbate intake was significantly associated with a higher risk of CIN 2+ (including CIN 2, CIN 3, and invasive cervical cancer) after adjustment for high-risk HPV infection [124]. In contrast, another prospective cohort study involving 299,649 European females after a mean follow-up of nine years demonstrated no significant association between cervical neoplasia (including CIN 2, CIN 3 carcinoma in situ, and invasive cervical cancer) and ascorbate intake [125]. However, an increase in fruit intake of 100 g per day was significantly inversely associated with invasive cervical cancer [125]. Furthermore, a double-blind RCT with oral administration of 500 mg daily ascorbate to 141 females with minor cervical squamous atypia, or CIN I, demonstrated that ascorbate did not reverse this pathology after two years of treatment in these patients compared with the control group of female [126].

From a clinical perspective, detecting plasma ascorbate levels may be more likely to reflect the biological effects of ascorbate in the body than evaluating dietary intake and provide a more reasonable evaluation of the relationship between ascorbate and cervical cancer. There are scarce data involving the relationship between plasma ascorbate levels, CIN, and invasive cervical cancer. A study from South Korea showed that the plasma concentrations of ascorbate were significantly lower in 58 patients diagnosed with CIN (0.36 mg/dL) than in the control group (0.48 mg/dL) (*p* < 0.05) [121]. Another study of 46 untreated cervical cancer and premalignant patients from Brazil demonstrated that plasma ascorbate concentrations were lower in the cervical cancer group but not in patients with premalignant lesions compared with controls [120]. Analyses among 120 participants with invasive cervical cancer in Nigeria revealed a decreasing mean level of ascorbate with the increase in the stage of the disease, and all had significantly lower plasma ascorbate levels compared to control patients [127]. Overall, although some meta-analyses and prospective cohorts support the role of ascorbate in cervical carcinogenesis, there are no large-scale clinical trials to decisively support these results. In future trials, study designs should consider incorporating dynamic changes in high-risk HPV status and cervical cytology, as well as plasma ascorbate instead of FFQs, to increase the accuracy and reliability of the studies.

#### 4.3.2. The Antitumor Role of Ascorbate in Cervical Cancer

Several studies found that the treatment of cervical cancer cell lines (Hela and Siha) with ascorbate from 0.5 to 10 mM exhibited significant inhibition of cell proliferation [18,128,129]. As in other types of cancer cells, ascorbate inhibits cell growth through multiple mechanisms, including inducing cellular stress, mitochondrial dysfunction, and DNA damage and causing cell cycle arrest and exogenous and endogenous apoptosis in cervical cancer cells [18,128,129]. The ROS scavenger N-acetylcysteine at 5 mM selectively inhibited ROS production induced by 1 mM or 10 mM ascorbate in Hela cells, suggesting that ascorbate at the millimole level acts as a pro-oxidant to reduce cell proliferation [18]. Moreover, ascorbate downregulated the expression of the E6 protein and the HPV transcriptional modulator c-Jun/c-Fos AP-1 heterodimer and enhanced the expression of wild-type p53 in Hela cells [130]. Although some studies have shown that ascorbate has synergistic effects when combined with cisplatin or doxorubicin by increasing ROS levels and apoptotic activity in cervical cancer cells, the increased expression of wild-type p53 may also contribute to the synergistic effect through the p53 pathway [130,131]. In addition, a research study examining the impact of ascorbate in a mouse model of methylcholanthrene (MCA)-induced cervical cancer revealed that administering ascorbate for either the first 6 weeks or for the entire 16-week induction period resulted in a twofold decrease in cancer incidence, compared to mice receiving ascorbate only during the final 10 weeks [132]. Overall, these data suggest that ascorbate is a potential therapeutic agent in preclinical models of cervical cancer, which establishes an experimental basis for the future development of new therapeutic strategies for cervical cancer.

## 5. Conclusions

As an essential water-soluble vitamin to maintain hemostasis in the human body, ascorbate exhibits distinct effects in males and females due to inherent differences in body mass, sex hormones, and lifestyles, resulting in higher plasma ascorbate levels in females. High-dose ascorbate has been shown to be a potential therapeutic agent that inhibits cell proliferation, reduces invasive capacity, and acts synergistically with chemotherapeutic agents in preclinical models of ovarian, endometrial, and cervical cancers. More clinical trials are needed to investigate the effects of high-dose intravenous ascorbate in combination with cisplatin or paclitaxel in patients with ovarian cancer. Although epidemiological studies remain inconclusive on the efficacy of ascorbate in preventing ovarian and endometrial cancers, the role of ascorbate in reducing the risk of HPV infection and persistent HPV status, as well as affecting the progression of CIN and early cervical invasive cancer, requires further exploration in well-designed, large-scale clinical trials.

## Figures and Tables

**Table 1:** Recommended diet allowance (RDA) for ascorbate during the lifetime of human beings.

Age (Years)	RDA for Females	RDA for Males
1–3	15 mg *	15 mg
4–8	25 mg	25 mg
9–13	45 mg	45 mg
14–18	65 mg	75 mg
19–50	75 mg	90 mg
51–70	75 mg	90 mg
>71	75 mg	90 mg
Pregnancy	14–18 years: 80 mg	-
	19–50 years: 85 mg	
Lactation	14–18 years: 115 mg	-
	19–50 years: 120 mg	

*: /day of ascorbate. The data is from [28].

**Table 2:** Overview of ascorbate as a cofactor of oxygenases.

Physiological Process	Cofactor for Enzyme	Function of Enzyme	Ref.
Collagen production	Prolyl 4-hydroxylase (Three isoenzymes for collagen synthesis) Prolyl 3-hydroxylase Lysyl hydroxylase	Hydroxylation of procollagen to produce and secrete collagen	[55,56]
Carnitine synthesis	Trimethyllysine hydroxylase γ-Butyrobetaine hydroxylase	Two hydroxylation reactions in carnitine synthesis	[57]
Hormones synthesis	Dopamine β-monooxygenase Peptidyl-glycine α-amidating monooxygenase	Norepinephrine biosynthesis Amidation of peptide hormones (calcitonin, oxytocin, vasopressin, GLP-1, substance P, neuropeptide Y)	[1,58,59]
Amino acid metabolism	4-hydroxyphenylpyruvate dioxygenase	Tyrosine elimination	[60]
Gene regulation	Asparaginyl hydroxylase or Factor Inhibiting HIF (FIH) Prolyl 4-hydroxylase (Three isoenzymes for HIF)	HIF signaling pathway regulation	[61,62,63]
Epigenetic modifications	JmjC domain-containing histone demethylases lysine-specific histone demethylases Ten-eleven translocases (TETs) alkylated DNA repair protein AlkB homologs (AlkBHs) fat-mass and obesity-associated protein (FTO)	Histone demethylation Cytosine demethylation RNA demethylation	[64,65,66,67,68]

## Data Availability

No new data were created or analyzed in this study. Data sharing is not applicable to this article.

## Abbreviations

AMPK	AMP-activated protein kinase
ATM	Ataxia–telangiectasia mutated
BRCA	Breast cancer gene
CIN	Cervical intraepithelial neoplasia
CN	Cervical neoplasia
DHA	Dehydroascorbic acid
EMT	Epithelial–mesenchymal transition
FFM	Fat-free mass
FFQ	Food frequency questionnaire
HPV	Human papillomavirus
HR	Hazard ratio
IP	Intraperitoneal
IV	Intravenous
MCA	Methylcholanthrene
mTOR	Mechanistic target of rapamycin
OC	Oral contraceptives
OR	Odds ratio
OS	Overall survival
PARP	Poly(ADP ribose) polymerase
PFS	Progression-free survival
RCT	Randomized clinical trial
RDA	Recommended diet allowance
ROS	Reactive oxygen species
SNPs	Single-nucleotide polymorphisms
SVCT	Sodium-dependent vitamin C transporter
SVCT1	Sodium-dependent vitamin C transporter 1
SVCT2	Sodium-dependent vitamin C transporter 1
